# Image-based crop disease detection with federated learning

**DOI:** 10.1038/s41598-023-46218-5

**Published:** 2023-11-06

**Authors:** Denis Mamba Kabala, Adel Hafiane, Laurent Bobelin, Raphaël Canals

**Affiliations:** 1 INSA CVL, University of Orleans, PRISME Laboratory EA 4229, 88 Boulevard Lahitolle, 18000 Bourges, France; 2 INSA CVL, University of Orleans, LIFO Laboratory EA 4022, 88 Boulevard Lahitolle, 18000 Bourges, France; 3 University of Orleans, INSA CVL, PRISME Laboratory EA 4229, 12 Rue de Blois, 45067 Orléans, France

**Keywords:** Engineering, Mathematics and computing

## Abstract

Crop disease detection and management is critical to improving productivity, reducing costs, and promoting environmentally friendly crop treatment methods. Modern technologies, such as data mining and machine learning algorithms, have been used to develop automated crop disease detection systems. However, centralized approach to data collection and model training induces challenges in terms of data privacy, availability, and transfer costs. To address these challenges, federated learning appears to be a promising solution. In this paper, we explored the application of federated learning for crop disease classification using image analysis. We developed and studied convolutional neural network (CNN) models and those based on attention mechanisms, in this case vision transformers (ViT), using federated learning, leveraging an open access image dataset from the “PlantVillage” platform. Experiments conducted concluded that the performance of models trained by federated learning is influenced by the number of learners involved, the number of communication rounds, the number of local iterations and the quality of the data. With the objective of highlighting the potential of federated learning in crop disease classification, among the CNN models tested, ResNet50 performed better in several experiments than the other models, and proved to be an optimal choice, but also the most suitable for a federated learning scenario. The ViT_B16 and ViT_B32 Vision Transformers require more computational time, making them less suitable in a federated learning scenario, where computational time and communication costs are key parameters. The paper provides a state-of-the-art analysis, presents our methodology and experimental results, and concludes with ideas and future directions for our research on using federated learning in the context of crop disease classification.

## Introduction

Crop disease detection and management is an area where data science can provide valuable decision tools to improve productivity, reduce costs, and support development of more environmentally friendly crop treatment methods^1,2^. Precision agriculture, which relies on modern technologies to improve productivity, has revolutionized the way farmers manage their crops. One of the main goals of precision agriculture is to optimize crop yields while ensuring crop quality and environmental preservation^3^. This includes reducing the negative impact of pesticides and other plant protection products. To achieve this, it is essential to use appropriate technological solutions capable of automatically detecting leaf diseases^4^, which are often responsible for significant economic losses and reduced crop quality and yield.

Many systems have been developed for automatic detection and management of crop disease, making use of modern technologies such as artificial intelligence, big data, image processing, and machine learning algorithms. Advances in these technologies in the field of agriculture have opened up promising opportunities for early detection and diagnosis of crop abnormalities^5^. Deep neural networks, such as convolutional neural networks (CNNs) or recurrent neural networks (RNNs), as well as modern approaches based on attention mechanisms (e.g., Vision Transformers)^6,7^, are widely used, and have demonstrated outstanding performance in crop anomaly detection and diagnosis^8–10^.

The various machine learning algorithms used require collecting data, which is then stored on a central server for model training. However, collecting consistent data in large quantities is difficult, time-consuming, expensive, and impractical for producers. In addition, the centralized approach used to train these models presents several limitations, including privacy and data security issues for producers who must release their private data, limited availability of data for model training, regulatory constraints on data protection (such as GDPR : General Data Protection Regulation), prohibitive transfer costs in a context of exploding data volumes to be processed.

Federated learning is one promising approach to overcoming these challenges. Data used to train ML algorithms can reveal unique information on production techniques, crop disease detection, and management methods, or the effectiveness of different agricultural practices. As a result, they are considered sensitive by economic actors, and sharing them represents a competitive risk. By enabling several entities (growers) to collaborate or work together to train a model without sharing private data with a central server, federated learning guarantees data confidentiality and security, and also makes it possible to train models in remote or rural areas. Moreover, by minimizing sharing of personal data and giving users control over their data, federated learning reduces the risk of data leakage. It then ensures compliance with data privacy regulations, such as GDPR, and furthermore alleviates pressure on device resources. Each participant in the federated learning process ultimately benefits from a more robust and efficient model trained on a richer and wider range of data, regardless of the distribution and size of each participant’s dataset^11–13^. Despite the many advantages of federated learning, a number of challenges remain, including complexity of coordination, data heterogeneity, effective communication, and prevention of malicious attacks.

In this paper, we evaluated and studied convolutional network models and models based on attention mechanisms (in particular Vision Transformers, ViT) using federated learning for disease detection. We have used four open source image datasets from the “PlantVillage” platform for our experiments. Our goal is to highlight the strengths of federated learning in crop disease classification, particularly with respect to user data security and enforcement of confidentiality of sensitive data.

The article is structured as follows : Section "Related work" presents a state-of-the-art on crop disease detection and classification methods and federated learning. Section "Detection of crop diseases" describes the working methodology, going through the federated learning framework implemented, then the models and data used. Section "Federated learning (FL)" presents the results and analyses of our experiments with models in the federated learning approach. Finally, Section "Methodology" concludes the article by summarizing our findings and proposing perspectives for our future work in the context of crop disease classification using the federated learning approach.

## Related work

In this section, we summarize agricultural research into crop disease detection using machine learning algorithms and the Visual Transformer. Several recent works have used these models, either jointly or individually, following a centralized approach to data processing. Next, we introduce the federated learning approach, mentioning major contributions, and summarize recent studies in the agricultural sector for crop disease detection.

### Detection of crop diseases

Crop diseases, considered as an abnormal condition affecting growth, yield and quality of a plant, have been the subject of many diagnostic and detection approaches. In recent years, methodologies based on machine learning and deep learning have been developed to detect plant diseases^6,7,14–16^.

CNNs are prevalent in detecting plant diseases, as detailed in studies^5^ and^17^. Specifically^14^, employs VGG-16 and GoogleNet on rice plants, achieving accuracies of 92.24% and 91.28% after data augmentation^8^. Uses VGG-16 and AlexNet on tomato leaves, yielding 97.29% and 97.49% accuracy after hyperparameter tuning^18^. Introduces a new CNN architecture tested on various plants, outperforming several pre-trained models with accuracies up to 99.66% and fewer parameters^3^. showcases a Deep CNN for 13 plant types, obtaining 96.46% accuracy, surpassing many popular models and traditional algorithms. Collectively, these works affirm the effectiveness of CNNs in early and accurate plant disease detection.

While CNN-based models excel in computer vision tasks, including in agriculture, they often struggle with capturing long-term data dependencies due to their local receptivity. The Vision Transformer (ViT) model, introduced in^19^, addresses this by utilizing attention mechanisms, enabling it to focus on all parts of an image based on their relevance. This has resulted in remarkable results in leaf disease detection^6,7,20,21^, as ViT can process high-resolution images, understand global context, and benefit from extensive pre-training. In^6^, the authors integrate a CNN with ViT for plant disease classification, showing that combining attention with CNNs balances accuracy with prediction speed^7^. Offers a new method using data fusion and Transformer networks for detecting late blight in vineyards, leveraging the synergy of diverse data like satellite images and weather data through three Transformers.

Considering the different works mentioned above, it is established that in the agricultural domain, CNNs and ViT approaches offer promising results for leaf disease treatment, identification, detection and classification. However, all these studies are only based on a centralized approach for model training, and do not address the idea of a distributed approach with federated learning, which is currently considered as a solution for the development of more robust general models, guaranteeing data privacy and security.

### Federated learning (FL)

Federated learning was introduced in^22^ as a distributed machine learning approach. Its techniques aim to train algorithms on decentralized devices while keeping data private at the local level. This approach contrasts with traditional machine learning techniques that are centralized in nature, but also with techniques based on parallel computing. The latter are designed to optimize computation for machine learning on multiple processors using a centralized dataset. Although powerful, current machine learning techniques have data privacy shortcomings because they require sharing training data with a central server, which can pose privacy, security, and regulatory issues related to data collection and sharing^12,13^.

In federated learning, local participants (devices) train their local models based on their own private data. Updates to the local models are then sent to a central server, which aggregates them through an aggregation algorithm to form a global model^11^. Local participants update their local models according to the global model and repeat this process until convergence is achieved. Depending on the application scenarios, federated learning can be categorized into horizontal federated learning, vertical federated learning and transfer federated learning [Li2020]. To ensure data privacy and security, different aggregation techniques have been proposed^23–25^.

Recent studies on federated learning, including concepts, challenges, privacy and security, and future research directions have been conducted in^11,13,26^. Growing steadily in recent years, federated learning has been applied to solve different types of problems in several domains, including medical^27–29^, distributed networks and systems^25,30,31^, Internet of Things (IoT)^32^, and very recently in the agricultural domain^33–35^.

Healthcare systems have traditionally relied on centralized data sharing, which presents security vulnerabilities. Federated learning is emerging as a solution, offering more secure data sharing between hospitals and preserving data confidentiality. Among recent work in this area, in^27^, the authors examine the fusion of federated learning, artificial intelligence (AI) and explainable AI (XAI) for smart healthcare applications. They argue that this combination can address many challenges in healthcare. In^28^, the focus is on the use of federated learning in biomedical health informatics. The authors envision a system in which various sources of health data are interconnected, guaranteeing data confidentiality and subsequently improving the quality of care. The article^29^ highlights the integration of federated learning in intelligent healthcare systems, particularly for remote patient monitoring. They highlight the value of merging federated learning and IoT in smart hospitals, suggesting the training of local models on IoT devices to avoid data transfers to the cloud.

Federated learning, widely used in networks, distributed systems, and IoT due to vast data and security vulnerabilities, aims to ensure system functionality while minimizing attack risks. Paper^25^ introduces “FedProx”, a framework for network heterogeneity. Article^30^ reveals the superiority of “model poisoning” over “training data poisoning”. Study^31^ suggests methods to cut communication costs, ideal for bandwidth-limited devices. Lastly^32^, delves into federated learning’s impact on IoT, especially in smart health and transportation.

Agricultural data, dispersed over many devices, poses privacy and security challenges. Federated learning offers solutions to these challenges, although it’s an emerging area in agriculture. In^33^, a Privacy-Encoding-Based Federated Learning (PEFL) framework is introduced to enhance security in smart farming, proving efficient in identifying normal and attack patterns from IoT data^35^. Examines federated learning for corn disease prediction, indicating the AlexNet CNN model as efficient, with a direct relation between CNN parameters and data exchange volume during training. Meanwhile, in^34^ a study of federated learning based on drones (or UAV: Unmanned Aerial Vehicle) for plant disease diagnosis, including classification of different types of insect pests, is conducted. The authors performed preprocessing and data augmentation to improve the classification accuracy, achieving 99.55% accuracy for all nine classes with the CNN model.

In reviewing the various works on federated learning presented above, it is clear that the agricultural domain remains the least explored. Of the few studies that exist to date, while they address security issues to ensure privacy and safety, or disease detection, many issues remain unexplored. In particular, issues related to studying the impact of varying the number of local devices on model performance, studying the impact of varying the number of local iterations or epochs on model performance, or studying the impact of varying the number of communication rounds or rounds between local devices and the central server on model performance. None of the existing work fully exploits the various popular CNN models to determine which are best suited for this new paradigm. Moreover, despite the successes of models based on attention mechanisms, such as Transformers in vision tasks, no work on the application of federated learning in the agricultural domain addresses these models. In this work, we study these different issues through experiments on a problem of disease classification of some plants, in order to provide appropriate answers.

## Methodology

### Federated learning framework

**Figure 1:**
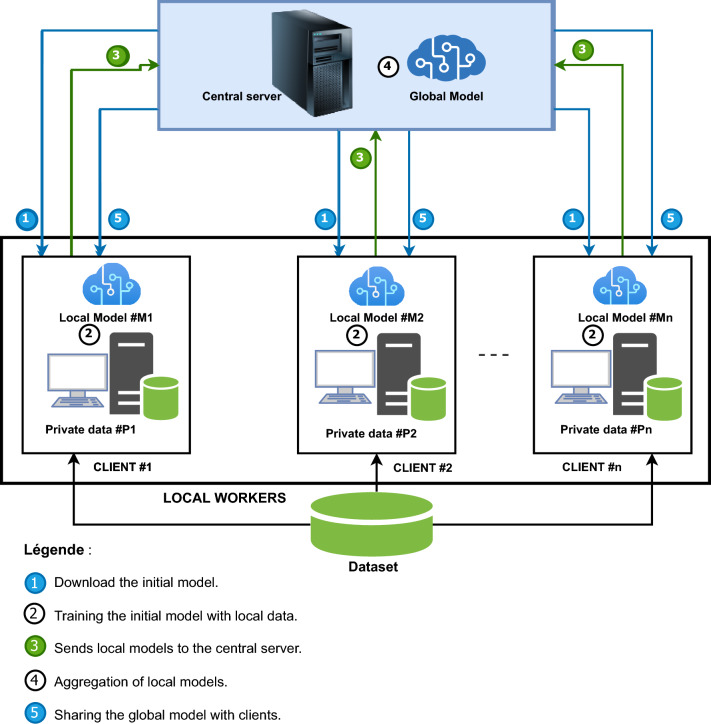
Working methodology.–We have a working environment comprising a central server for local model aggregation, and local workers with n clients for local model training.

Federated learning is a machine learning framework in which multiple workers or clients contribute to the training of a more robust and efficient global model that they will later share while keeping their data decentralized^11^. Figure 1 shows an overview of the deployed FL architecture. We used open source datasets from the “PlantVillage” platform, and then simulate a collaborative set of clients who contribute to the improvement of a global model.

The adopted methodology, detailed in Fig. 1, is built around five essential phases. Phase 1, **initialization**, involves setting up a pre-trained model on a server that is then distributed to various pre-defined clients in the environment. During phase 2, **local training**, each client use the received model to perform local training based on their individual plant leaf data, this data coming from the training data subset. Then in phase 3, **parameter transfer**, in which updates to the local models, once training is complete, are sent to the central server for aggregation, while all client training data are kept undisclosed on their local devices. In phase 4, **model aggregation**, the various parameters of the local models are aggregated on the central server using a chosen aggregation algorithm, generating an updated global model. Finally, Phase 5, **the global model transfer and local evaluation**, involves sharing the consolidated, improved, and updated global model with the individual clients for the next iteration, and then evaluating it on the test data subset.

This five-phase cycle is repeated until the predefined number of communication rounds between the central server and the individual clients participating in the process is reached. Thus, in each communication round, an evaluation of the overall model is performed by each client on its own test data. Once the final model is ready, it is distributed to all clients in the environment to perform inferences. These different steps defines the behavior ot two main roles : the Client role (local workers) and the Server role (central federated server). Algorithms 1 and 2 describe more formally the behavior each of these two roles.

**Algorithm 1:**
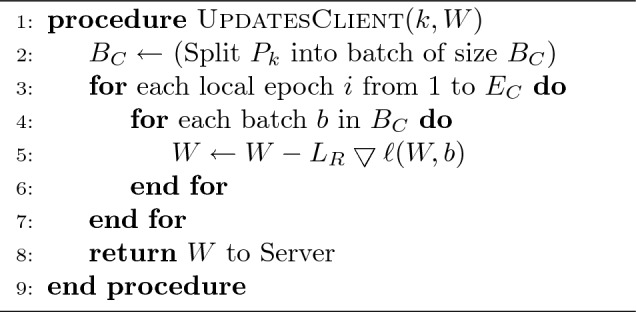
Client_Role

**Algorithm 2:**
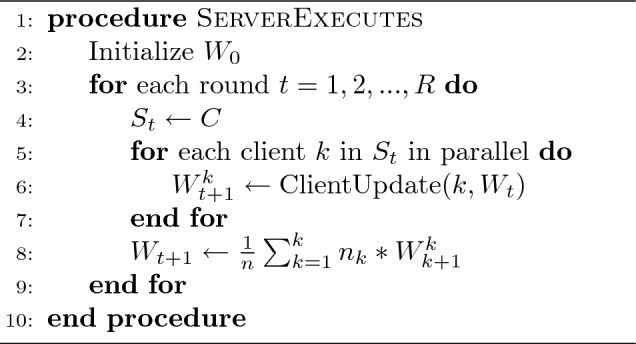
Server_Role

In these two algorithms *k* denotes the index of *C* clients; $B_{c}$ denotes the size of the local batches; $P_{k}$ denotes the dataset partition for client *k*; $E_{c}$ denotes the number of iterations performed by the local models; *W* denotes the model weights; $L_{R}$ denotes the local learning rate, and *R* denotes the number of communication rounds between the central server and the clients.

#### Client process (ClientUpdate)

This defines the behavior of the different clients in the environment, as well as how they update the initial and later the global model using their local data. In this process, some parameters are initially fixed, namely: the total number of clients $C=\sum _{i=1}^{C}k_{i}$; the number of communication rounds $R=\sum _{i=1}^{R}r_{i}$; the number of epochs $E_{c} = \sum _{i=1}^{E}e_{i}$; the local learning rate $L_{R}$; and the size of local batches $B_{c}$. We therefore vary these different parameters during our experiments in order to observe the impact of each of them in the different model’s performances. To do so, we perform a simulation of a collaborative set of clients who share a dataset with the same characteristics (consisting of healthy and infected leaves) for training and testing the model. The data partitioning of the dataset among the different clients is performed equally and randomly distributed. In other words, $D = \sum _{k=1}^{n} P_{k}$, where *D* denotes the size of the dataset, with $P_{k}(k=1, 2,..., n)$ the partition of each client, and $P_{1}=P_{2}=...= P_{n}$.

#### Server process

This is the federated learning process that takes place at the central server. This process is essentially in charge of collecting the different local models and then aggregating them in order to obtain a global aggregated model that will be shared with the clients at the end of the communication cycle. The aggregation is achieved through the utilization of an *aggregation algorithm*. This algorithm serves as the logical mechanism at the central server level to merge the updates from the local models involved in the training cycle. Federated averaging is the commonly used approach to aggregation. The following is a non-exhaustive list of some of the more well-known aggregation algorithms:

- **Federated Averaging (FedAvg)** Proposed in^22^, FedAvg consists in aggregating local models by taking a weighted average of the weights of each model to obtain a global model. In this approach, only the model parameters are shared and not the raw data. This is one of the most popular federated learning algorithms.
- **Federated Proximal (FedProx)** Presented in^25^, FedProx is an improved version of FedAvg that addresses non-IID (Non-Identical and Independently Distributed) and data heterogeneity problems. This algorithm reduces the impact of devices with very different data or unbalanced data distributions.
- **Federated Stochastic Gradient Descent (Federated SGD)** This is a variant of the stochastic gradient descent (SGD) algorithm^24^. The federated SGD uses different weights for each of the local models based on the corresponding performance, and the aggregation is done by taking a weighted average. Unlike FedAvg, Federated SGD does not require devices to perform multiple local training epochs before sharing their model updates.
- **Secure Multi-Party Computation Averaging (SMC-Avg)** Presented in^23^, this aggregation algorithm is based on Secure Multi-party Computation (SMC). The idea of SMC is to allow multiple parties to compute a function on their inputs while preserving the confidentiality of the latter. In the context of federated learning, SMC-Avg is used to aggregate model updates securely without revealing individual device updates.

These different algorithms play a key role in any federated learning topology and aim to improve the privacy of local model updates. In our case, we used the federated averaging algorithm (FedAvg). The aggregation procedure is well described in Algorithm 2, part “Server Process”.

### Models and datas

In this subsection, we will discuss the issue related to the different models used, as well as the data collection used for our experiments.

#### Deep learning architectures

During our different experiments, we used seven pre-trained models. These models were chosen for their common use in the literature as well as for their remarkable performances. Among them, five are of the CNN type and two are of the transform type.

- **VGG-16** Developed by Oxford’s Visual Geometry Group^36^, VGG-16 is a deep learning model based on the AlexNet architecture. Unlike AlexNet, VGG-16 uses smaller 3$\times$3 convolution filters, reducing the number of parameters.
- **ResNet50** Introduced in^37^, ResNet50 is a 50-layer convolutional neural network based on the VGG architecture (VGG-16 and VGG-18). It forms networks by stacking residual blocks. Its original design allows more layers to be added without encountering the problem of gradient leakage, thanks to a residual bottleneck block.
- **DenseNet-121** DenseNet networks^38^ are convolutional neural network characterized by dense connections between its layers. This architecture stacks dense blocks that concatenate inputs from previous layers, maintaining identical spatial resolution.
- **MobileNet-V2** Developed by Google^39^, MobileNet is a convolutional neural network designed for mobile and embedded applications. It uses depth-separable convolutions to create lightweight, low-latency networks, optimizing efficiency and speed while minimizing the number of parameters.
- **Inception-V3** An evolution of Google’s Inception-V1 model^40^, Inception-V3 is a deep convolutional neural network architecture. It features improvements such as label smoothing, factorized convolutions and an auxiliary classifier.
- **Visual Transformer (ViT)** Introduced in 2021^19^, ViT is a visual model based on the architecture of transformers originally designed for natural language processing. Applying attention to sequences of image patches, it directly predicts class labels for the image.

#### Data description

For training and validation of the FL architecture, we used publicly available data from the PlantVillage database. This contains over 50,000 images of healthy and infected plant leaves, divided into 38 categories according to species and diseases. It is a repository of images on plant health, freely accessible and allowing the development of disease diagnostics.

In our case, we used data from four types of plants for our different experiments: grapes, apples, corn, and tomatoes. These data are classified according to different categories of healthy and infected leaves. A description of the latter is provided in Table 1.

**Table 1:** Data description.

Dataset label	Class label	Number of images
Grape	Black_rot	4062
	Black_Measles	
	Leaf_blight	
	Healthy	
Apple	Apple_scab	3161
	Black_rot	
	Cedar_apple_rust	
	Healthy	
Corn	Leaf_spot	3852
	Common_rust	
	Healthy	
	Leaf_blight	
Tomato	Bacterial Spot	18.160
	Early Blight	
	Late Blight	
	Leaf Mold	
	Septoria leaf spot	
	Two Spotted Spider Mite	
	Target Spot	
	Yellow Leaf Curl Virus	
	Mosaic Virus	
	Healthy	

Description of the data used in our work according to leaf classes and number of images per dataset.

**Figure 2:**
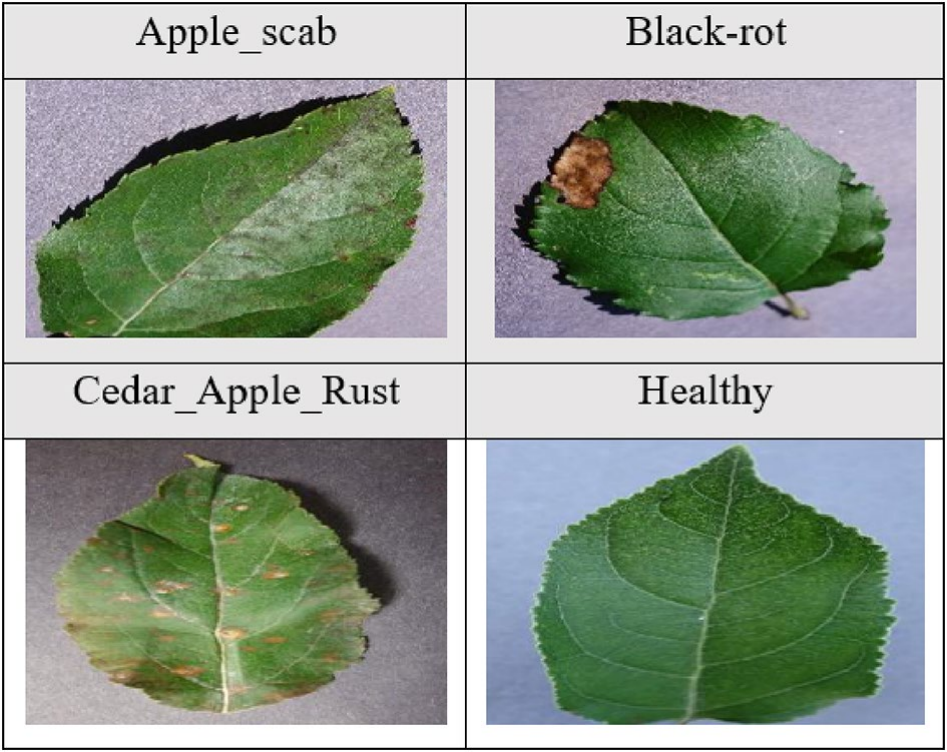
Images of four classes of Apple leaves.— Extract of four images of plant leaves from the Apple dataset.

**Figure 3:**
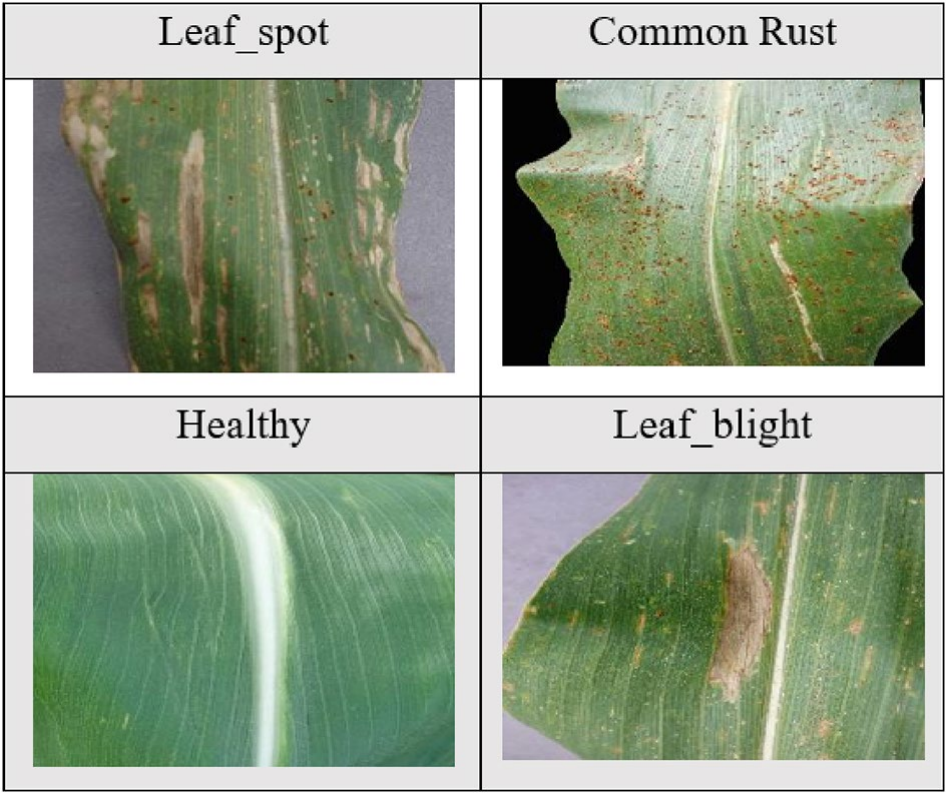
Images of four classes of Corn leaves.—Extract of four images of plant leaves from the Corn dataset.

**Figure 4:**
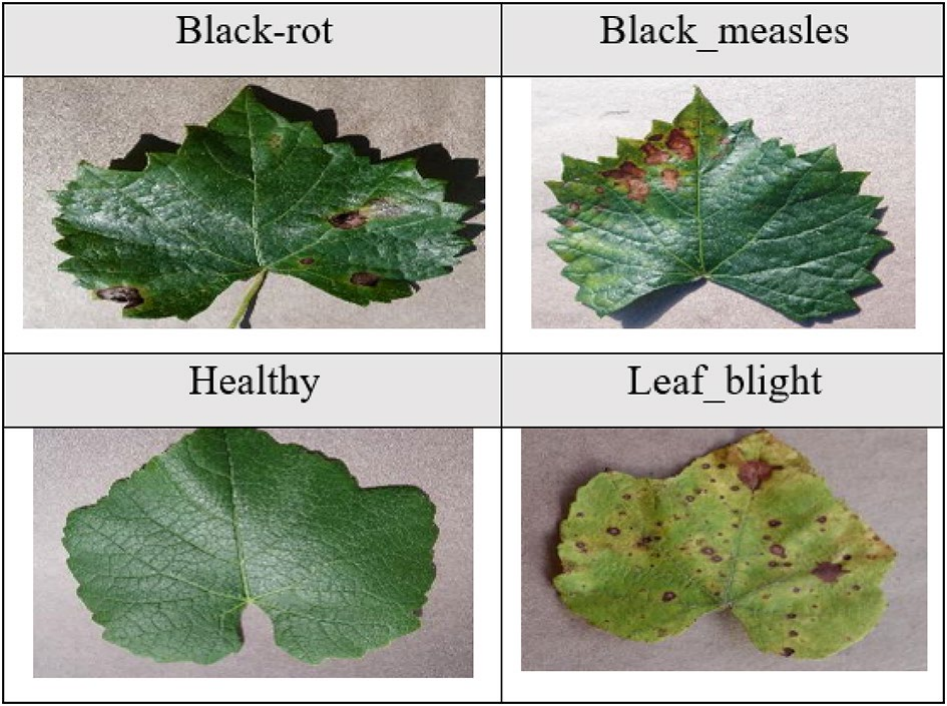
Images of four classes of Grape leaves.– Extract of four images of plant leaves from the Grape dataset.

**Figure 5:**
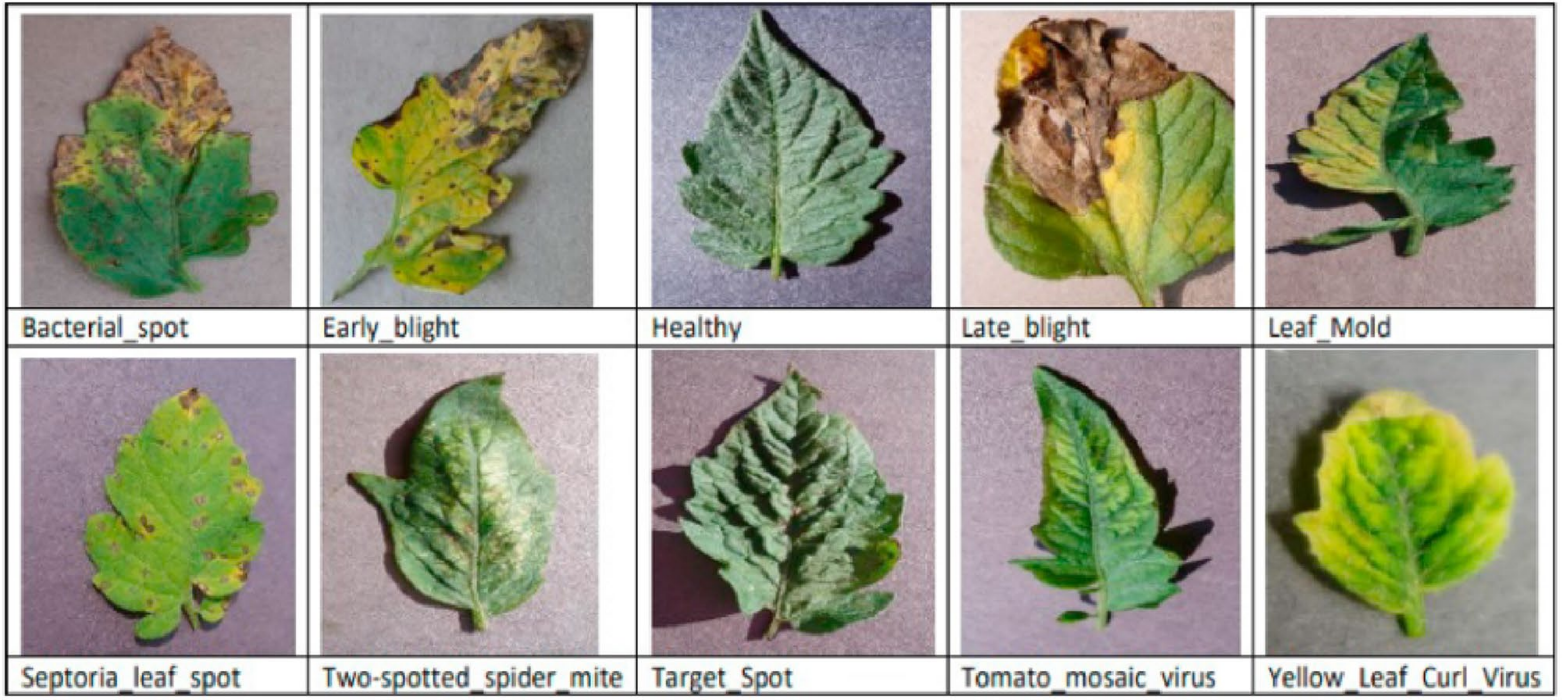
Images of ten classes of Tomato leaves.– Extract of ten images of plant leaves from the Tomato dataset.

Below are some images of the leaves corresponding to the different classes of each of the four plants. Figure 2 shows images of apple leaves, Figure 3 shows images of corn leaves, Figure 4 shows images of grape leaves, and Fig. 5 shows images of tomato leaves.

The accuracy of deep learning models depends largely on the quality, quantity, and contextual meaning of the training data. Before using our images, we applied some transformations to them to improve the performance and results of our models. Although there are several image transformation techniques, they operate either on the position or on the color of the images. In our case, we performed two operations: resizing the input images to the size (224 $\times$ 224) so that they all have a consistent shape, and normalizing them using the standard approach, with the mean and standard deviation, expressed as follows: $Z = (X-m) / \sigma$, where *Z* represents the normalized data, *X* the input data, *m* the empirical mean, and $\sigma$ the standard deviation. Given the mean (*mean*[1], ..., *mean*[*n*]) and standard deviation (*std*[1], ..., *std*[*n*]) for the *n* channels, this transformation will normalize each channel of the input. The main idea through these transformations is to have training and test data sets with similar distributions in order to guarantee a good generalization of our models.

## Experiments and results

In this section, we present the different experiments conducted in our study. Thus we give below the experimental configuration of our development environment, the different measures used to evaluate the performance of our models, as well as the results obtained and their interpretation for each experimental scenario.

### Experimental setup

The experiments were carried out using Python 3.9.12, on a computer equipped with a 3.60 GHz Intel Xeon W-2223 processor and an NVIDIA TU104GL Quadro RTX 5000 graphics card, running Ubuntu 20.04.4 LTS 64 bits. We used Pytorch and Keras libraries for developing Deep Learning architectures, as well as cuDNN 8.2.1 and the CUDA 11.2.67 toolkit for deep learning on the GPU. We ran the experiments on four datasets, varying the parameters defined in Table 2 for each of them.

**Table 2:** Experimental parameters.

	Client	Epoch/client	Batch size	Rounds	Classes	Learning rate
Values	3, 5, 7	1, 5	16	10, 30, 50, 100	4, 10	0.01

We have set values for the key parameters to be used in our experiments.

### Performance evaluation metrics

We used four metrics to assess the performance of our different models, namely precision, recall, accuracy, and F1-score. These metrics are essential for understanding the strengths and weaknesses of our models, and they take into account indices based on the number of true positives (TP), true negatives (TN), false positives (FP), and false negatives (FN)^41^.

- **Accuracy** It provides an overall estimate of the proportion of correct predictions in relation to all predictions. It is expressed as follows: $Accuracy = (TP+TN)/(TP+FP+TN+FN)$
- **Precision** It measures the ability of a model to not classify a negative sample as positive. It is expressed as follows: $Precision = TP/(TP+FP)$
- **Recall** It measures the ability of a model to identify all positive samples. It is given as follows: $Recall = TP/(TP+FN)$
- **F1-Score** This is the harmonic mean of precision and recall. It measures the global performance of a model by taking into account both precision and recall, but also by giving a compromise between the two. It is expressed as follows: $F1-Score = 2*(Recall*Precision)/(Recall+Precision)$

As we proceed, we will use only two measures to present the performance of the models studied, namely F1-Score and Accuracy.

### Presentation of results

In this section, we present the results of different tests of the models for the different parameters, consisting in the variation of the number of clients participating in the federated learning process to train the models, the variation in the number of communication rounds between the clients and the server, the variation in the number of epochs at the local level for each client, and finally a comparative study of the performance of the models according to the four datasets of the PlantVillage platform selected for experiments. We evaluate the performance of the selected models with respect to four specific aspects :

- The influence of the number of clients,
- The influence of the number of communication rounds between clients and the server,
- The influence of the number of iterations or epochs locally for each client,
- The comparison of the performances with respect to the different datasets.

For the first three experimental scenarios, the results presented below refer to the vine leaf dataset, while the results for the fourth scenario correspond to a synthesis of our four datasets. For each of these scenarios, we used the same configuration using the parameters defined in Table 2.

#### Influence of the number of clients

We performed this experiment in order to analyze the impact of varying the number of clients on the performance of models using the decentralized federated learning approach during training. We examined three different configurations of participants (3, 5, and 7 clients), while keeping the same number of communication rounds (Rounds = 30) between the clients and the server, as well as the same number of local iterations for each client (epoch = 1). Table 3 presents the performance results of the different deep architectures for the three participant configurations mentioned above.

**Table 3:** Performance of models in relation to the number of clients.

	Client =3		Client =5		Client =7	
	F1-Score	Accuracy	F1-Score	Accuracy	F1-Score	Accuracy
ResNet50	**99,59**	**99,59**	**99,24**	**99,23**	**99,52**	**99,52**
DenseNet121	98,38	98,39	97,32	97,32	96,88	96,89
Vgg16	98,68	98,68	97,50	97,49	96,96	96,94
MobileNetV2	98,59	98,58	97,79	97,79	97,54	97,55
InceptionV3	92,57	92,77	80,71	84,57	80,13	83,86
ViT-B16	96,95	96,94	96,01	96,00	94,83	94,80
ViT-B32	95,72	95,69	94,77	94,77	92,93	92,95

We successively increase the number of participants by 3, 5 and 7, keeping rounds=30 and epoch=1 constant.

Significant values are in [bold].

From the results obtained in Table 3, we can observe that the ResNet50 model outperforms all tested models, regardless of the client configuration, with Accuracy and F1-Score averages close to 99.5%. The MobileNetV2, Vgg16, and DenseNet121 models also perform well, but slightly worse than ResNet50, with Accuracy and F1-Score averages close to 98%, 97.7%, and 97.5%, respectively, for the different client configurations. The Vision Transformer architecture variants ViT_B16 and ViT_B32 underperforms than ResNet50, MobileNetV2, Vgg16, and DenseNet121, with Accuracy and F1-Score averages of 95.9% and 94.5 respectively for ViT_B16 and ViT_B32 for different client configurations. The InceptionV3 yields poorer results compared to other models, with an Accuracy and F1-Score of about 92% for 3 clients.

**Figure 6:**
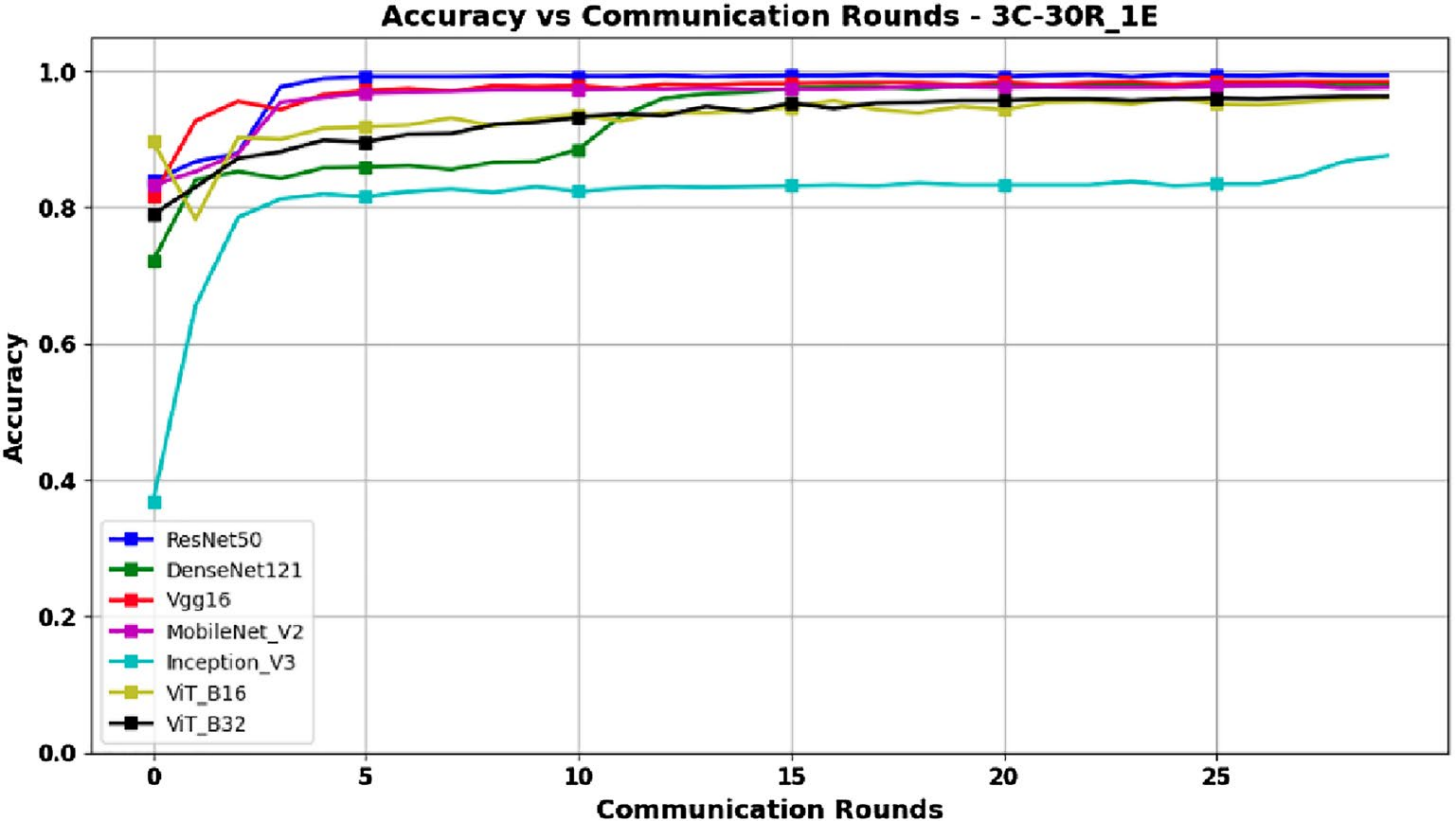
Accuracy versus Communication Rounds for 3 clients.–This graph shows the Accuracy of models in the configuration where number of clients=3, rounds=30 and epoch=1.

**Figure 7:**
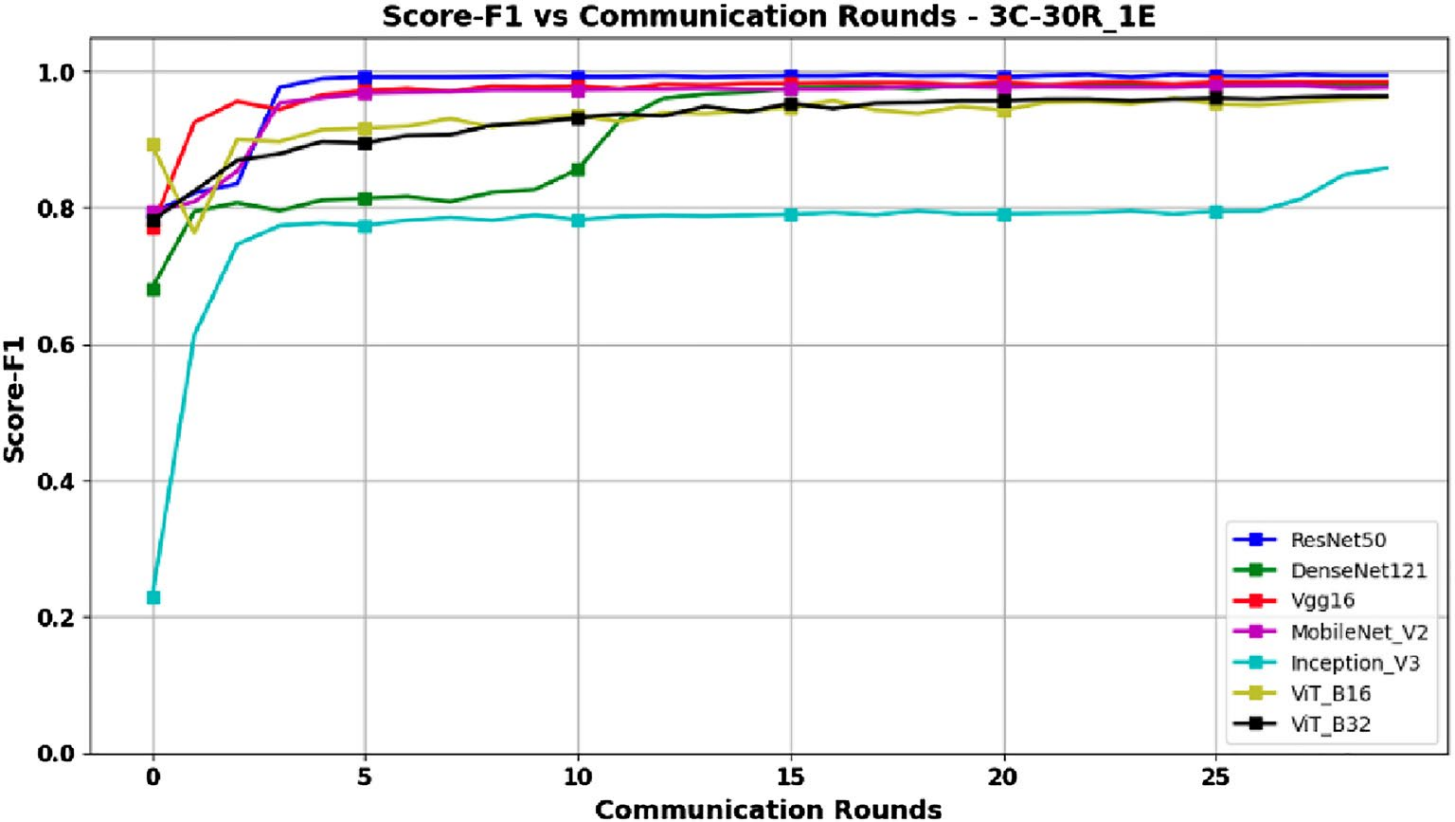
F1-Score versus Communication Rounds for 3 clients.— This graph shows the F1-Score of models in the configuration where number of clients=3, rounds=30 and epoch=1.

Based on the data presented in Table 3, along with the graphical representations in Figs. 6 and 7 depicting Accuracy and F1-Score respectively, it can be deduced that the ResNet50 architecture, despite its complexity, delivers remarkable results. These outcomes indicate that ResNet50 offers a compelling advantage by significantly reducing training costs, making it an optimal choice within this experimental context. The graphs in Figs. 6 and 7 also show that ResNet50 achieves better performance than other models after only 5 rounds, thus reducing the training time, and consequently reducing the energy consumption, which is an important advantage in the context of federated learning.

The MobileNetV2 is specifically designed for applications where energy consumption is critical, i.e., to reduce the computational costs associated with the training phase. Although its performance is slightly lower than ResNet50 for different client configurations, MobileNetV2 is still an attractive choice if a lighter, more cost-effective model with competitive performance. Although Vgg16 and DenseNet121 are also complex, they may be viable alternatives if one is looking to reduce complexity slightly compared to ResNet50, while still achieving satisfactory performance. On the other hand, ViT_B16 and ViT_B32 have a high computational complexity due to the use of attention mechanisms, and require a significant amount of memory. In view of the results obtained, they are not necessarily the most efficient options in a context where resources are limited and computation time is a priority.

**Figure 8:**
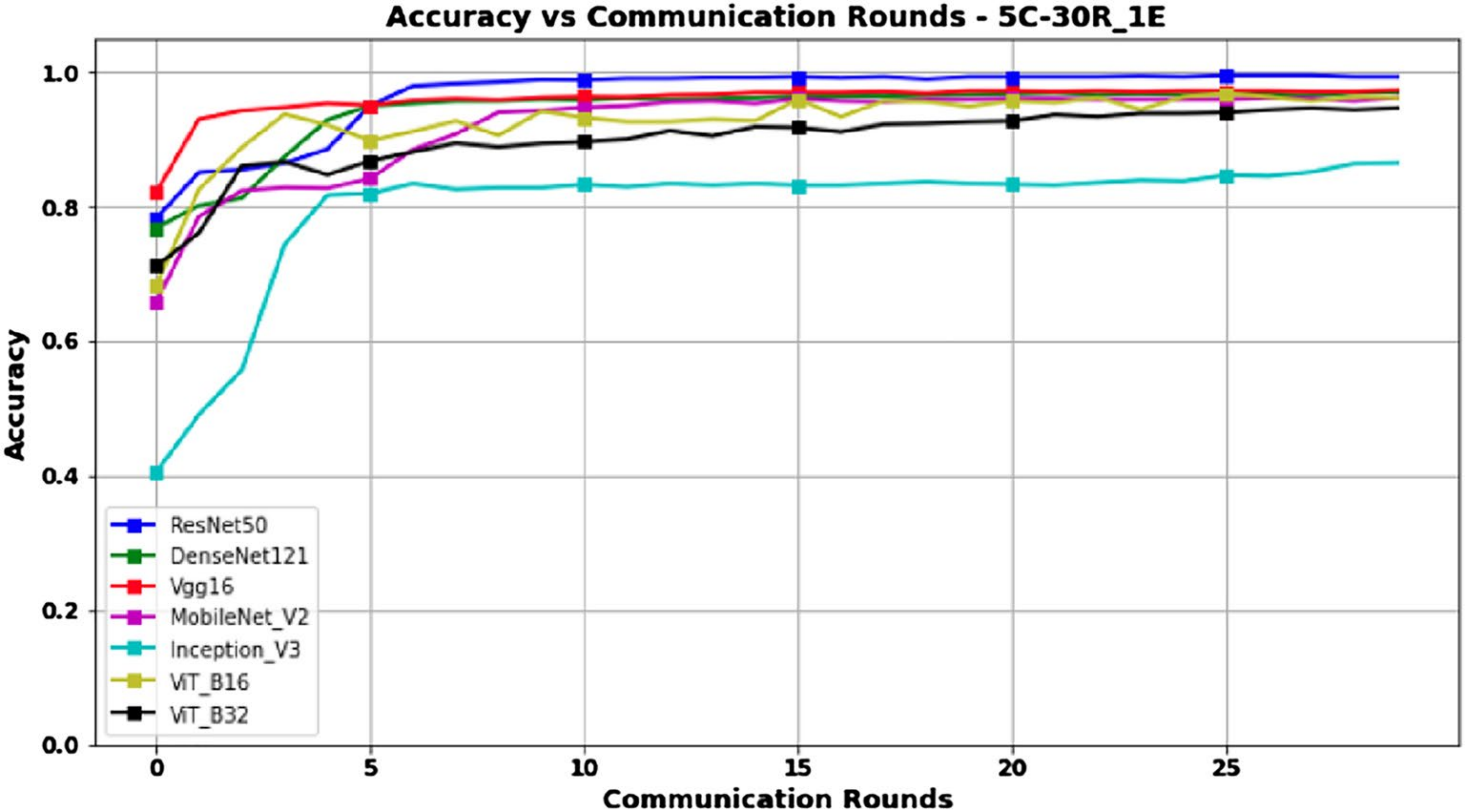
Accuracy versus Communication Rounds for 5 clients.— This graph shows the Accuracy of models in the configuration where number of clients=5, rounds=30 and epoch=1.

**Figure 9:**
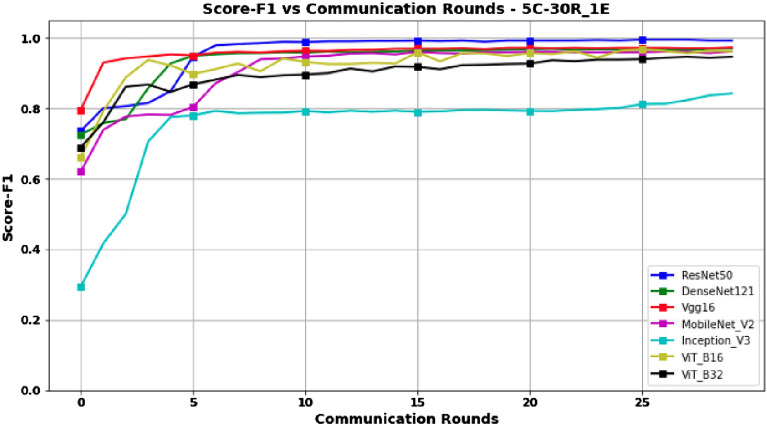
F1-Score versus Communication Rounds for 5 clients.— This graph shows the F1-Score of models in the configuration where number of clients=5, rounds=30 and epoch=1.

Unlike the 3-client setup, the results obtained in the 5-client configuration, as presented in Table 3 and Figs. 8 and 9 for Accuracy and Score-F1, respectively, show that although ResNet50 is still the best model in terms of performance compared to the others; performances of all models tend to decrease slightly. This decrease can be explained by the fact that, while still having the same amount of data overall, increasing the number of clients leads to a decrease in the amount of data for each client, and thus the model has less data to train compared to the 3-client configuration. Furthermore, unlike the other models, Figures 8 and 9 show that the performance of the InceptionV3 model decreases significantly as the number of clients increases.

**Figure 10:**
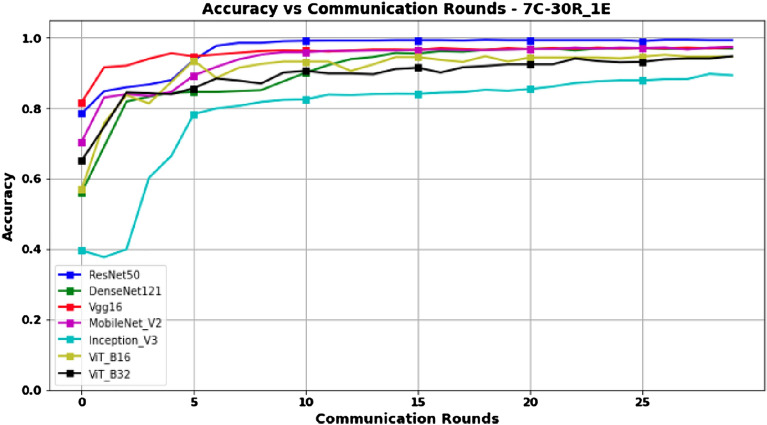
Accuracy versus Communication Rounds for 7 clients.—This graph shows the Accuracy of models in the configuration where number of clients=7, rounds=30 and epoch=1.

**Figure 11:**
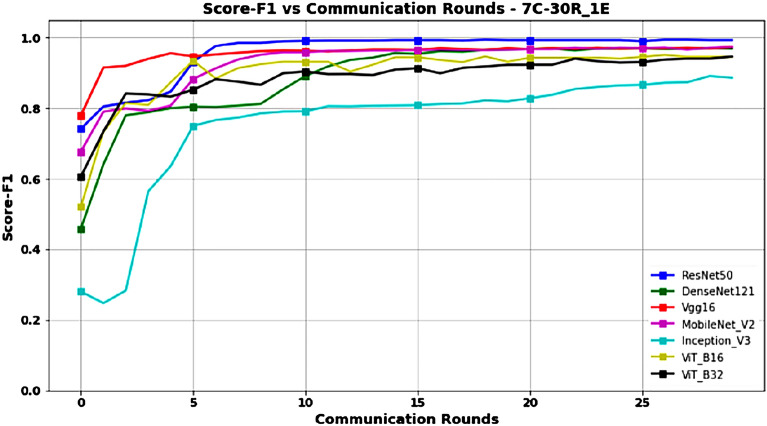
F1-Score versus Communication Rounds for 7 clients.— This graph shows the F1-Score of models in the configuration where number of clients=7, rounds=30 and epoch=1.

Figures 10 and 11 represent Accuracy and Score-F1 in a 7-client configuration, respectively. Except for the ResNet50 model, which scales better with an increase in the number of clients, the performance of all other models is lower than that obtained in the 3- and 5-client configurations, probably due to the decrease in the amount of data available for training.

From this experiment, we find that overall and from the results obtained, the FL model training process is impacted by the number of clients involved, although the magnitude of this impact varies with each model’s architecture. The ResNet50, MobileNetV2, Vgg16, and DenseNet121 models tend to see a decrease in performance as the number of clients increases, although this decrease is relatively small for ResNet50 and MobileNetV2. In other words, these two models demonstrate more resilience towards scaling up the number of clients in federated learning model training. Therefore, ResNet50 and MobileNetV2 are better choices for this federated learning scenario involving an increasing number of clients, especially for the plant leaf classification task. On the other hand, the performance of InceptionV3, ViT_B16, and ViT_B32 decreases more rapidly with the increasing number of clients, which means that these architectures are less suitable for the FL model training approach in this specific type of scenario. On the other hand, the performance of InceptionV3, ViT_B16 and ViT_B32 decreases more rapidly as the number of clients increases, meaning that these architectures do not seem to be a suitable choice for a FL configuration with a large number of clients and less training data. A compromise should therefore be found between the number of clients and the amount of training data.

#### Impact of the communication rounds

The objective of this experiment is to study the impact of increasing the number of rounds on model performance. The rounds correspond to the number of model’s parameters exchanges between each client and the aggregation server. We considered four setups where the number of rounds successively takes the values 10, 30, 50 and 100. For the number of participants in the process, we chose the 3-client configuration, as it offers a good trade-off between performance and the amount of data in terms of partition, as described in the previous subsection. Table 4 shows the results of the experiment based on the specifications indicated above, and considering one epoch (epochs=1) for each round of a given client.

**Table 4:** Performance of the models in relation to the number of rounds.

	Round=10		Round=30		Round=50		Round=100	
	F1-Score	Accuracy	F1-Score	Accuracy	F1-Score	Accuracy	F1-Score	Accuracy
ResNet50	**99,10**	**99,11**	**99,59**	**99,59**	**99,47**	**99,47**	**99,52**	**99,52**
DenseNet121	96,37	96,38	98,38	98,39	97,93	97,92	97,97	97,98
Vgg16	96,83	96,84	98,68	98,68	97,24	97,24	97,87	97,86
MobileNetV2	96,32	96,31	98,59	98,58	97,38	97,37	99,04	99,04
InceptionV3	79,74	83,49	92,57	92,77	91,76	92,22	94,60	94,61
ViT-B16	91,86	91,93	96,95	96,94	96,23	96,22	98,47	98,44
ViT-B32	91,96	91,93	95,72	95,69	97,37	97,37	97,74	97,72

We successively increase the number of rounds by 10, 30, 50 and 100, keeping the number of clients=3 and epoch=1 constant.

Significant values are in [bold].

Analyzing the results presented in Table 4, we generally found that increasing the number of rounds improves the performance of most models. ResNet50 achieves the best performance for each round scenario, with a maximum F1-Score and Accuracy value of 99.59% achieved at only 30 rounds. The DenseNet121, Vgg16, and MobileNetV2 models also show a trend of improving performance as the number of rounds increases. Although, they fail to match ResNet50, nevertheless, they achieve satisfactory performance, with maximum F1-Score and Accuracy values of about 98.4% and 98.7% for DenseNet121 and Vgg16 at 30 rounds, and 99.04% for MobileNetV2 at 100 rounds, respectively. Although performing worse than all other models studied in all scenarios, InceptionV3 shows an improvement in performance with increasing number of rounds, from a F1-Score of 79.74% and Accuracy of 83.49% for 10 rounds to a F1-Score of 94.60% and Accuracy of 94.61% for 100 rounds. ViT_B16 and ViT_B32 also show a general trend of improvement in performance as the number of rounds increases, with maximum F1-Score and Accuracy values close to 98.5% and 97.7% respectively for ViT_B16 and ViT_B32 at 100 rounds.

**Figure 12:**
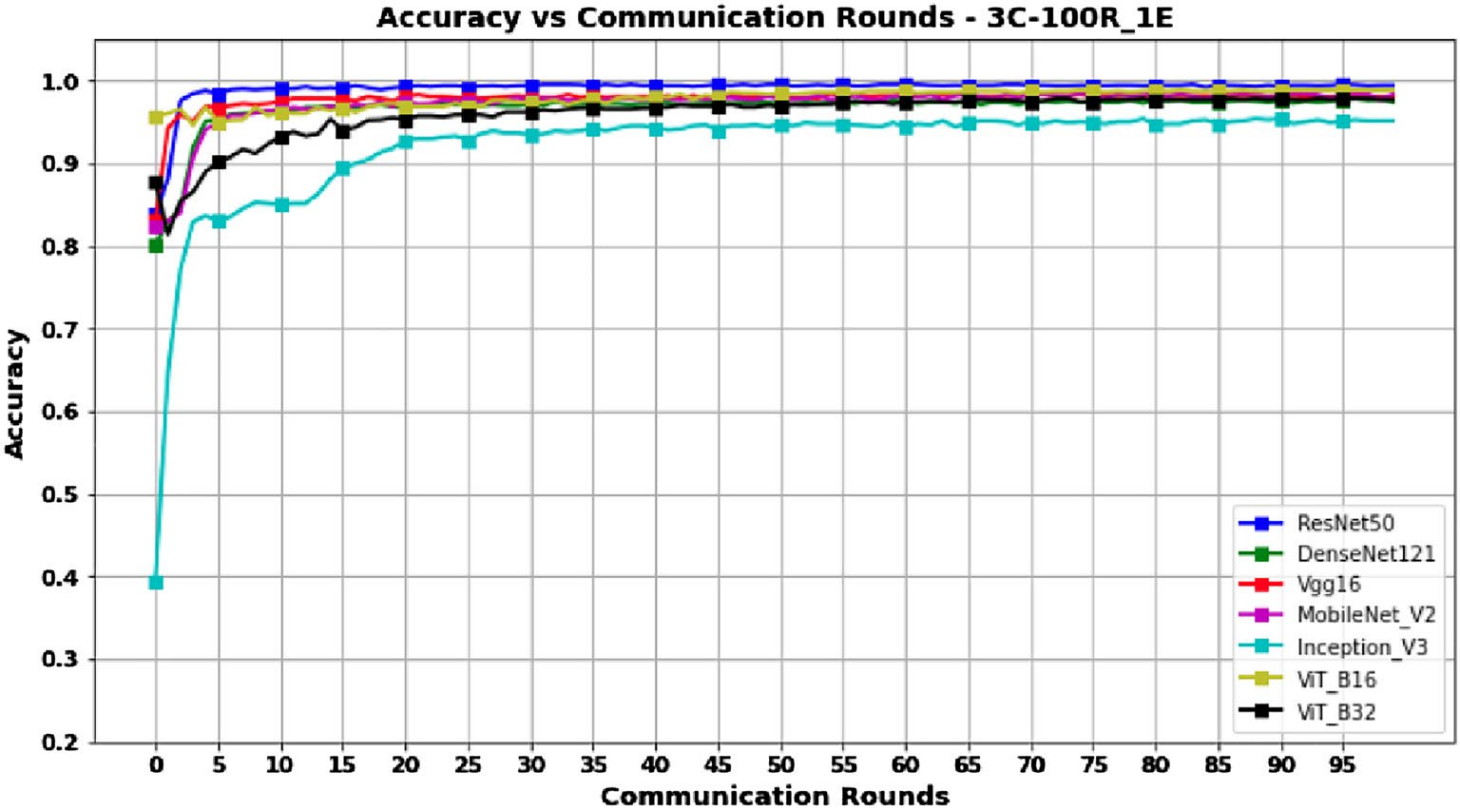
Accuracy versus Communication Rounds for 3 clients.—This graph shows the Accuracy of models in the configuration where number of clients=3, rounds=100 and epoch=1.

**Figure 13:**
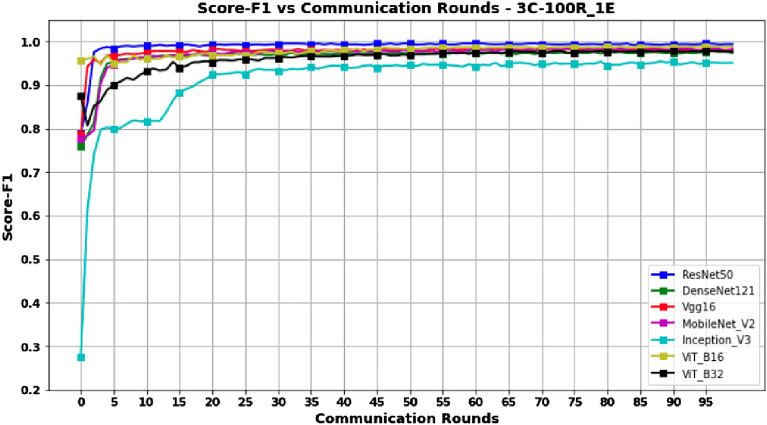
F1-Score versus Communication Rounds for 3 clients.—This graph shows the F1-Score of models in the configuration where number of clients=3, rounds=100 and epoch=1.

Looking at the results in Table 4, it can be seen in Figs. 12 and 13 that the ResNet50, DenseNet121, and Vgg16 models reach their peak performance after only 30 rounds, with no significant improvement with a higher number of rounds. Thus, these models may be a wise choice to minimize communication costs while maintaining high performance. In contrast, for InceptionV3, ViT_B16, and ViT_B32, their maximum performance is reached at 100 rounds, which implies higher communication costs and may make them less preferable in terms of the tradeoff between performance and communication cost. In particular, MobileNetV2 also reaches its peak performance at 100 rounds, with a Score-F1 and Accuracy of 99.04%. While this also incurs high communication costs, it may be an acceptable choice if maximum performance is required despite the additional communication cost.

Taking into account the complexity of the basic architectures of each model studied as well as the communication costs, ResNet50 provides very good balance between performance, communication cost, and model complexity. It reaches its peak performance with a relatively small number of rounds (30 rounds) while maintaining high performance and moderate complexity. The DenseNet121 model, known for its complex architecture^38^, also achieves good performances, close to that of ResNet50. However, its complexity may lead to an increase in computational resources, which may be acceptable in exchange for high performance and a relatively efficient communication cost. Vgg16 and MobileNetV2 have simple and lightweight architectures^36,39^, and thus are less complex than the other models studied. In a context where there are computational and communication resource constraints, and where one accepts slightly lower performance than ResNet50, these models might be a good choice, although the number of rounds required for MobileNetV2 is higher. Regarding InceptionV3, being the most complex^40^ of the CNNs studied, it might not be the appropriate choice when considering both communication costs and model complexity. Finally, based on Transformers^19^, and considering the obtained performances, ViT_B16 and ViT_B32 might be less preferable compared to the other studied models due to their higher complexity and communication costs.

#### Effects of the local iterations

In this section, we studied the impact of increasing the number of iterations or epochs locally for each client participating in the FL process for model training. Indeed, since the training process in FL is distributed across multiple devices, the number of epochs corresponds to the number of complete iterations on the training dataset on each client. Thus, for this experiment, we first consider a scenario where the epochs parameter is set to 1, with 3 clients and 50 rounds. Then, keeping the same configuration (3 clients and 50 rounds), we increased the value of epochs to 5 in order to compare the corresponding performance for these two scenarios. Table 5 shows the experimental results based on the specifications.

**Table 5:** Model performance versus number of epochs.

	Epochs=1		Epochs=5	
	F1-Score	Accuracy	F1-Score	Accuracy
ResNet50	**99,47**	**99,47**	**99,76**	**99,76**
DenseNet121	97,93	97,92	99,02	99,04
Vgg16	97,24	97,24	98,74	98,75
MobileNetV2	97,38	97,37	99,11	99,11
InceptionV3	91,76	92,22	96,82	96,81
ViT-B16	96,23	96,22	98,15	98,15
ViT-B32	97,37	97,37	98,75	98,75

We successively increase the number of epochs by 1 and 5, keeping the number of clients=3 and rounds=50 constant.

Significant values are in [bold].

Looking at the results in Table 5, we generally observe an improvement in performance for all models studied when the number of local epochs is increased from 1 to 5. The ResNet50 model offers the best performance among all the other models tested, with a F1-Score of 99.47% and Accuracy of 99.76% for epochs=1, and 99.76% and 99.76% for epochs=5. While offering the best performance, increasing the number of epochs slightly improves performance for ResNet50. This suggests that it is a solid and efficient choice for this classification task, regardless of the number of epochs locally.

DenseNet121 and MobileNetV2 also perform slightly worse than ResNet50. DenseNet121 obtains a F1-Score of 97.93% and an Accuracy of 97.92% for epochs=1, and 99.02% and 99.04% for epochs=5. MobileNetV2 gets a F1-Score of 97.38% and an Accuracy of 97.37% for epochs=1, and 99.11% and 99.11% for epochs=5. Although they benefit more from the increased number of epochs than ResNet50 in terms of performance improvement, these two models may be a good alternatives in a context where a trade-off must be made between better performance and more computation time for local training.

The ViT_B32, Vgg16 and ViT_B16 models also achieve good performance, although lower than the ResNet50, DenseNet121 and MobileNetV2 models. When the number of epochs increases to 5, these models significantly improve their performance, with an almost identical F1-Score and Accuracy of 98.75% for Vgg16 and ViT_B32, and a F1-Score and Accuracy of 98.15% for ViT_B16. This suggests that these models require more local computation time to learn more about the data to improve their performance. The InceptionV3 model performed worst compared to the other architectures studied, with a F1-Score of 91.76% and Accuracy of 92.22% for epochs=1, and 96.82% and 96.81% for epochs=5. This model might not be a good choice in the context where computation time and quality of accuracy in classification are priorities.

**Figure 14:**
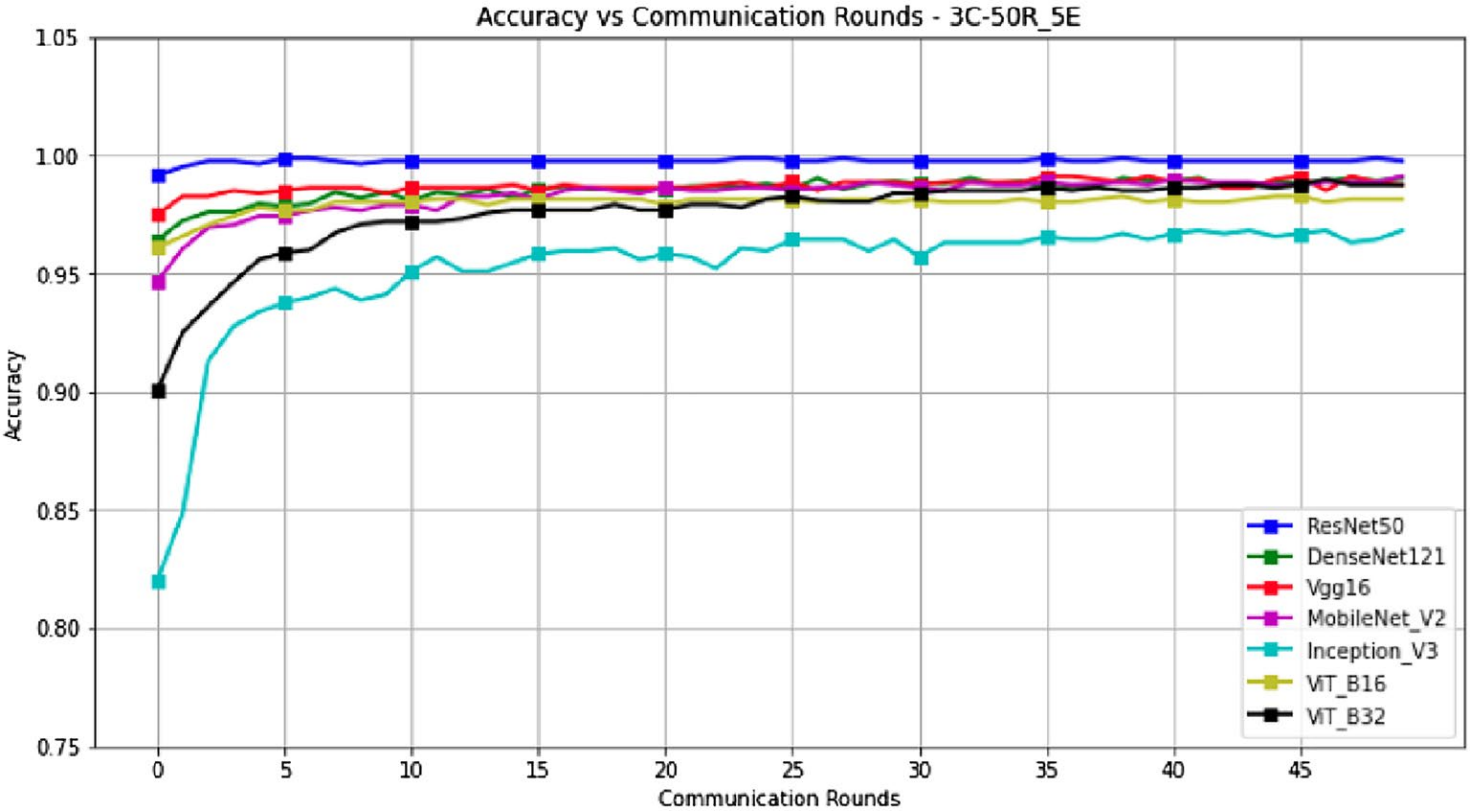
Accuracy versus Communication Rounds for 3 clients.—This graph shows the Accuracy of models in the configuration where number of clients=3, rounds=50 and epoch=5.

**Figure 15:**
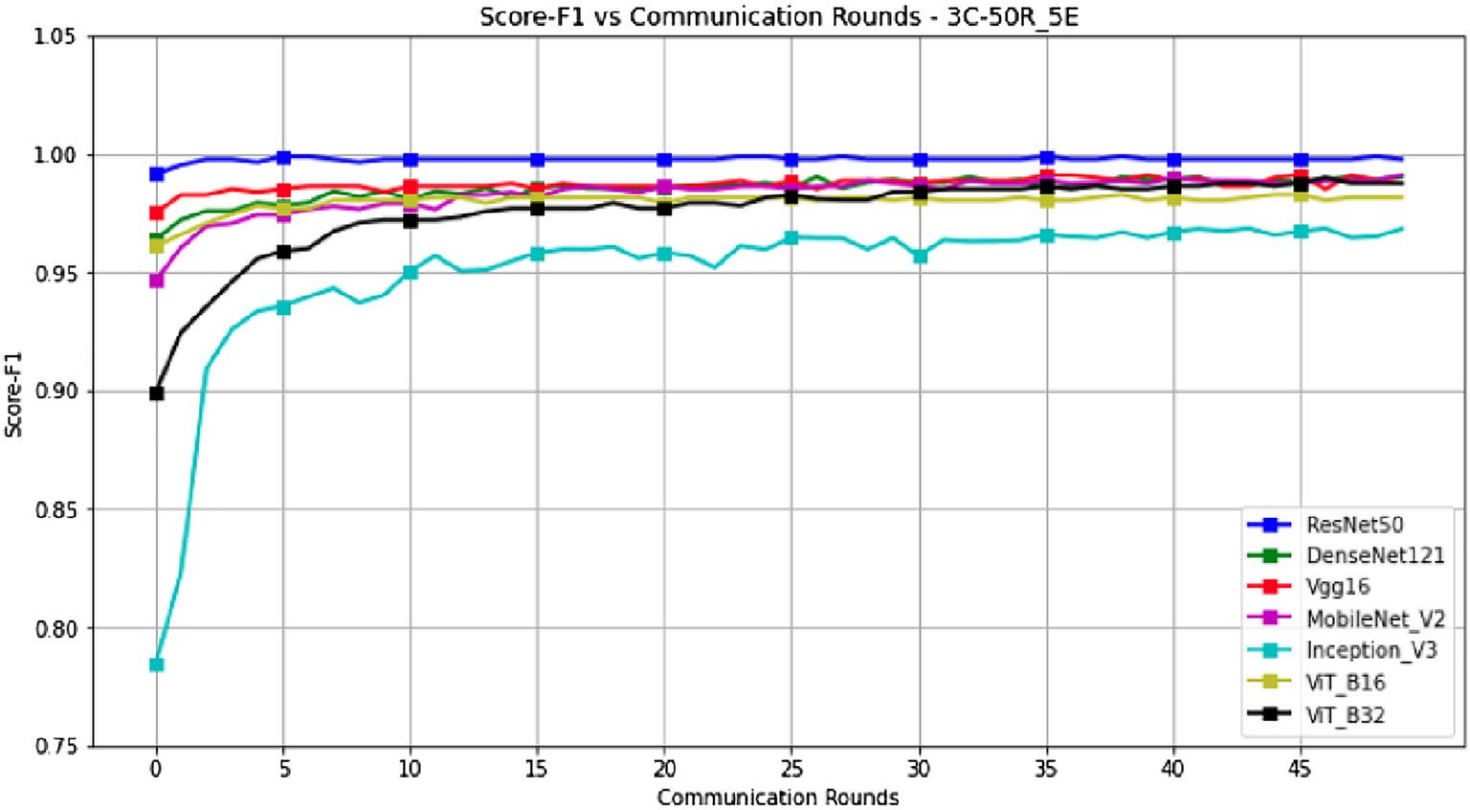
F1-Score versus Communication Rounds for 3 clients.— This graph shows the F1-Score of models in the configuration where number of clients=3, rounds=50 and epoch=5.

Figures 14 and 15 represent the evolution of Accuracy and F1-Score in a scenario where epochs=5, with a configuration of 3 clients and 50 rounds. These results are presented in Table 5.

In FL approach, it is crucial to take into account the communication cost and response time. Some models require more local computation time for training, which would make them an unwise choice. For example, although InceptionV3 experiences a significant performance improvement by increasing the number of epochs (with a difference in Score-F1 and Accuracy of 5.06% and 4.59%, respectively, for a change from epochs=1 to epochs=5), it would not be recommended in this context. MobileNetV2 is designed to be computationally efficient while maintaining high performance, making it a desirable alternative in a context where computation time is a major concern, even though its performance may be slightly lower than ResNet50. DenseNet121 and Vgg16 also experience an improvement in performance with the increase in the number of epochs, but to a lesser extent compared to InceptionV3, ViT_B16, and MobileNetV2. This implies that these two architectures could also be an acceptable choice, offering a good compromise between performance and computation time.

From this experiment, it is clear that increasing the number of epochs for each client locally generally improves the performance of all models studied. The ResNet50 model offers the best performance among all models. It does not seem to be impacted by the variation in the number of epochs as much as the other models, and is then the best choice in the context of FL, offering a better trade-off between performance and computation time. MobileNetV2, DenseNet121, and Vgg16 are also desirable alternatives in the context of a tradeoff between performance and computation time. Increasing the number of epochs, while having a positive impact on model performance, will increase the computational time required to train the models. It would therefore be ideal to determine the optimal convergence point, where the performance improvement no longer justifies the increase in computation time. In other words, it would be desirable to find a balance between performance and computational efficiency, especially when computational time is a priority.

#### Performance analysis across datasets

In this section, we conduct a comparative performance study of the different models studied for each of the four datasets from the PlantVillage platform, namely: Apple, Corn, Grape and Tomato. Table 6 summarizes the performance results of the models tested on our four datasets for a 3-client, 30-round configuration. Note that for these experiments, we used the same configuration with the parameters defined in Table 2 for each of the datasets.

**Table 6:** Performance of models against datasets.

	Apple		Corn		Grape		Tomato	
	F1-Score	Accuracy	F1-Score	Accuracy	F1-Score	Accuracy	F1-Score	Accuracy
ResNet50	**99,23**	**99,22**	**98,12**	**98,08**	**100,00**	**100,00**	**95,77**	**96,72**
DenseNet121	97,47	97,49	94,36	93,71	97,85	97,86	90,39	91,39
Vgg16	98,04	98,09	95,36	95,43	98,19	98,20	89,78	90,77
MobileNetV2	97,45	97,47	97,36	97,36	98,10	98,10	88,97	89,95
InceptionV3	83,60	87,05	92,20	93,11	96,11	96,20	80,08	82,57
ViT-B16	98,27	98,28	93,90	94,23	98,57	98,56	92,57	93,57
ViT-B32	97,76	97,81	94,91	95,07	98,22	98,22	91,19	92,21

We successively use each of our 4 datasets with an identical configuration, i.e. clients=3, rounds=30 and epoch=1.

Significant values are in [bold].

We can observe from the results presented in Table 6 that the performance of the models varies depending on each dataset. In general, we can notice that all models obtain better performances on the Grape dataset, while the performances are worse on the Tomato dataset. The ResNet50 model presents the best performances among all the models, whatever the dataset considered. Indeed, it manages to reach a F1-Score and an Accuracy of 100% on the Grape dataset. The ViT_B16 model also performs well, slightly less than ResNet50, but slightly better than the other models on the Grape, Apple and Tomato datasets. More precisely, it obtains respectively a F1-Score and Accuracy of 98.57% and 98.56% on the Grape dataset, a F1-Score and Accuracy of 98.57% and 98.56% on the Apple dataset, and finally a F1-Score and Accuracy of 92.57% and 93.57% on the Tomato dataset.

The Vgg16 and ViT_B32 models show almost similar performance on the Grape, Apple and Corn datasets. However, these performances are slightly lower than those of ResNet50 and ViT_B16. The F1-scores and Accuracies obtained by the two models are close to 98.2% for the Grape dataset, 98% for the Apple dataset, and 95% for the Corn dataset respectively. MobileNetV2 and DenseNet121 also perform well, varying by dataset, and slightly worse in most cases than ResNet50, ViT_B16, Vgg16 and ViT_B32. The InceptionV3 model performs poorly overall, compared to the other models tested, regardless of the dataset. InceptionV3 performs worse than all other models, but peaks on the Grape dataset with a F1-Score and Accuracy of 96.11% and 96.20% respectively.

We found that the majority of models exhibit superior performance, with very high F1-Score and Accuracy on the Grape dataset. This could mean that the images in this dataset are easier to classify, by the simple fact that they are overall sharper and have a better representation of the different classes. The performance of the models on the Tomato dataset is generally the poorest, compared to the other datasets. Although these images are of lower quality than the Grape dataset, it is clear that they are more difficult for the models to classify. Since the size of the Tomato dataset and the number of classes (10 classes) are significantly larger than those of the other datasets, the poor performance of the deep architectures on this dataset could be explained by the fact that the distribution of examples between classes is less balanced, thus making the classification task more difficult. It could also be due to the fact that the models need more examples to learn the discriminative features of the different classes of tomato leaves. Since the size and number of classes for this dataset is larger, we believe that tuning the hyperparameters taking into account the size of this dataset could improve the performance of the models.

The Apple and Corn datasets have the same number of classes (4 classes) and almost similar sizes as the Grape dataset. Due to the simpler distribution of classes and the size of these datasets, the deep architectures benefit from fast learning, which justifies the good performance of the models on these datasets, unlike the Tomato dataset. However, the variation in performance on the Grape, Apple, and Corn datasets could be due to the difference in data quality between these datasets. Thus, we have models that might be more robust to variations in data quality, while others are not. As an example, apart from the ResNet50 model, we can see through the results in Table 6 that the vision model ViT_B16 (see also ViT_B32) is only slightly impacted by data variations by offering slightly lower performances than ResNet50 compared to the other models for most datasets. The robustness of these models in this case is certainly explained by the use of attention mechanism. This mechanisms allow this type of model to capture information at different scales and levels of abstraction, thus improving their ability to learn more general and abstract visual features from the input images.

Compared with the results obtained by the CNNs in^3,8,18^, as well as the models based on attention mechanisms in^20,21^ which used the centralized approach and the “PlantVillage” platform datasets, we note that these results are close to those obtained in our work with the FL decentralized approach. In other words, in addition to its many advantages, FL is capable of performing the same tasks and obtaining results almost identical to those obtained with the centralized approach.

To summarize, the performance of the deep architectures varies according to each dataset. The quality of the data between the different datasets has a significant impact on the performance of the models. Differences in size and number of classes between datasets can also explain variations in model performance. Therefore, adjusting the hyperparameters of the models according to the size and number of classes of each dataset could optimize their performance.

## Conclusion

The use of machine learning technologies, in particular those based on Convolutional Neural Networks (CNNs) and Vision Transformers (ViTs), for crop disease classification is an evolving field. Thus, in this research, we explored the application of federated learning (FL) in this context, a new paradigm that has been underexplored in this field despite its numerous advantages regarding the security of user data and the preservation of confidentiality of sensitive data. Several important partial conclusions were drawn from the experiments conducted. First, the performance of the models trained through the FL process is influenced by the number of clients involved, with ResNet50 and MobileNetV2 proving to be more robust to an increase in the number of clients and more suitable for a federated learning scenario. Second, the number of communication rounds has an impact on the performance of the deep architectures, where ResNet50 proved to be an optimal choice for a balance between performance, computational cost, and model complexity. In addition, increasing the number of epochs locally for each client generally improves the performance of all models studied, with ResNet50 providing the best performance. The ViT_B16 and ViT_B32 Vision Transformers, while offering better performance than some CNNs, require more computational time, making them less suitable in a FL scenario. Finally, the performance of the deep models varies with each dataset, with significant differences depending on the quality of the data and the number of classes in each dataset.

It is clear that federated learning has a crucial role to play in crop disease classification. The results of this study highlight the importance of a well-designed and executed FL approach that takes into account the number of clients, the number of communication rounds, the number of local iterations, and the data quality. The issue of balancing performance and computational efficiency is a critical topic to consider, especially when computation time is a priority. Furthermore, it is crucial to choose the machine learning model architecture that best suited for FL scenario, as demonstrated by the efficiency of ResNet50 in several experiments. This research lays the foundation for a better understanding of the application of FL in crop disease classification. Further research is therefore needed to refine these findings and explore other aspects of FL in crop disease classification.requirements.

## Data Availability

The data we used for our experiments is part of a selection of over 50,000 images. These images, representing healthy and infected plant leaves, come from the public online platform “PlantVillage”. You can find them at PlantVillage dataset.

## References

[1] Aktar W, Sengupta D, Chowdhury A (2009). Impact of pesticides use in agriculture: Their benefits and hazards. Interdiscip. Toxicol..

[2] Chapter 7: Crop disease and agricultural productivity. In *Agricultural Productivity and Producer Behavior* 217–250. (University of Chicago Press, 2019). 10.7208/chicago/9780226619941.003.0008 . 10.7208/chicago/9780226619941.003.0008

[3] Geetharamani G, Pandian J. A (2019). Identification of plant leaf diseases using a nine-layer deep convolutional neural network. Comput. Electr. Eng..

[4] Ouhami M, Hafiane A, Es-Saady Y, Hajji ME, Canals R (2021). Computer vision, IoT and data fusion for crop disease detection using machine learning: A survey and ongoing research. Remote Sens..

[5] Tugrul B, Elfatimi E, Eryigit R (2022). Convolutional neural networks in detection of plant leaf diseases: A review. Agriculture.

[6] Borhani Y, Khoramdel J, Najafi E (2022). A deep learning based approach for automated plant disease classification using vision transformer. Sci. Rep..

[7] Maillet W, Ouhami M, Hafiane A (2023). Fusion of satellite images and weather data with transformer networks for downy mildew disease detection. IEEE Access.

[8] Rangarajan AK, Purushothaman R, Ramesh A (2018). Tomato crop disease classification using pre-trained deep learning algorithm. Procedia Comput. Sci..

[9] Sapkal, A. T. & Kulkarni, U.V. Comparative study of Leaf Disease Diagnosis system using Texture features and Deep Learning Features. https://www.ripublication.com/ijaer18/ijaerv13n19_39.pdf

[10] Kerkech M, Hafiane A, Canals R (2020). Vine disease detection in UAV multispectral images using optimized image registration and deep learning segmentation approach. Comput. Electron. Agric..

[11] Zhang C, Xie Y, Bai H, Yu B, Li W, Gao Y (2021). A survey on federated learning. Knowl. Based Syst..

[12] Yang Q, Liu Y, Chen T, Tong Y (2019). Federated machine learning. ACM Trans. Intell. Syst. Technol..

[13] Li T, Sahu AK, Talwalkar A, Smith V (2020). Federated learning: Challenges, methods, and future directions. IEEE Signal Process. Mag..

[14] Yakkundimath R, Saunshi G, Anami B, Palaiah S (2022). Classification of rice diseases using convolutional neural network models. J. Inst. Eng. India Ser. B.

[15] Nishad MAR, Mitu MA, Jahan N (2022). Predicting and classifying potato leaf disease using k-means segmentation techniques and deep learning networks. Procedia Comput. Sci..

[16] Kerkech M, Hafiane A, Canals R (2018). Deep leaning approach with colorimetric spaces and vegetation indices for vine diseases detection in uav images. Comput. Electron. Agric..

[17] Li L, Zhang S, Wang B (2021). Plant disease detection and classification by deep learning—A review. IEEE Access.

[18] Hassan SM, Maji AK (2022). Plant disease identification using a novel convolutional neural network. IEEE Access.

[19] Dosovitskiy, A., Beyer, L., Kolesnikov, A., Weissenborn, D., Zhai, X., Unterthiner, T., Dehghani, M., Minderer, M., Heigold, G., Gelly, S., Uszkoreit, J. & Houlsby, N. An image is worth 16x16 words: Transformers for image recognition at scale (2020). http://arxiv.org/abs/2010.11929

[20] Chen R, Qi H, Liang Y, Yang M (2022). Identification of plant leaf diseases by deep learning based on channel attention and channel pruning. Front. Plant Sci..

[21] Wang Y, Chen Y, Wang D (2022). Convolution network enlightened transformer for regional crop disease classification. Electronics.

[22] McMahan, H. B., Moore, E., Ramage, D., Hampson, S. & Arcas, B. A. y. Communication-efficient learning of deep networks from decentralized data (2016). http://arxiv.org/abs/1602.05629

[23] Bonawitz, K., Ivanov, V., Kreuter, B., Marcedone, A., McMahan, H. B., Patel, S., Ramage, D., Segal, A. & Seth, K. Practical secure aggregation for federated learning on user-held data (2016). http://arxiv.org/abs/1611.04482

[24] Lian, X., Zhang, C., Zhang, H., Hsieh, C.-J., Zhang, W. & Liu, J. Can decentralized algorithms outperform centralized algorithms? A case study for decentralized parallel stochastic gradient descent (2017). http://arxiv.org/abs/1705.09056

[25] Li, T., Sahu, A. K., Zaheer, M., Sanjabi, M., Talwalkar, A. & Smith, V. Federated optimization in heterogeneous networks (2018). http://arxiv.org/abs/1812.06127

[26] Mothukuri V, Parizi RM, Pouriyeh S, Huang Y, Dehghantanha A, Srivastava G (2021). A survey on security and privacy of federated learning. Futur. Gener. Comput. Syst. FGCS.

[27] Rahman A, Hossain MS, Muhammad G, Kundu D, Debnath T, Rahman M, Khan MSI, Tiwari P, Band SS (2022). Federated learning-based ai approaches in smart healthcare: Concepts, taxonomies, challenges and open issues. Clust. Comput..

[28] Xu J, Glicksberg BS, Su C, Walker P, Bian J, Wang F (2021). Federated learning for healthcare informatics. J. Healthc. Inform. Res..

[29] Mishra A, Saha S, Mishra S, Bagade P (2023). A federated learning approach for smart healthcare systems. CSI Trans. ICT.

[30] Bagdasaryan, E., Veit, A., Hua, Y., Estrin, D. & Shmatikov, V. How to backdoor federated learning (2019).

[31] Konečnỳ, J., McMahan, H. B., Yu, F. X., Richtárik, P., Suresh, A. T. & Bacon, D. Federated learning: Strategies for improving communication efficiency (2017).

[32] Nguyen, D. C., Ding, M., Pathirana, P. N., Seneviratne, A., Li, J. & Poor, H. V. Federated learning for internet of things: A comprehensive survey (2021). http://arxiv.org/abs/2104.07914

[33] Kumar P, Gupta GP, Tripathi R (2022). PEFL: Deep privacy-encoding-based federated learning framework for smart agriculture. IEEE Micro.

[34] Khan, F. S., Khan, S., Mohd, M. N. H., Waseem, A., Khan, M. N. A., Ali, S. & Ahmed, R. Federated learning-based UAVs for the diagnosis of plant diseases. In *2022 International Conference on Engineering and Emerging Technologies (ICEET)*. (IEEE, 2022). 10.1109/iceet56468.2022.10007133 .

[35] Antico, T., Moreira, L. & Moreira, R. Evaluating the potential of federated learning for maize leaf disease prediction. In *Anais do XIX Encontro Nacional de Inteligência Artificial e Computacional*, 282–293. (SBC, Porto Alegre, RS, Brasil, 2022). 10.5753/eniac.2022.227293 . https://sol.sbc.org.br/index.php/eniac/article/view/22789

[36] Simonyan, K. & Zisserman, A. Very deep convolutional networks for large-scale image recognition (2014). http://arxiv.org/abs/1409.1556

[37] He, K., Zhang, X., Ren, S. & Sun, J. Deep residual learning for image recognition (2015). http://arxiv.org/abs/1512.03385

[38] Huang, G., Liu, Z., Maaten, L. V. D., Weinberger, K .Q. Densely connected convolutional networks. In *2017 IEEE Conference on Computer Vision and Pattern Recognition (CVPR)*. (IEEE, 2017). 10.1109/cvpr.2017.243 .

[39] Howard, A. G., Zhu, M., Chen, B., Kalenichenko, D., Wang, W., Weyand, T., Andreetto, M. & Adam, H. MobileNets: Efficient convolutional neural networks for mobile vision applications (2017). http://arxiv.org/abs/1704.04861

[40] Szegedy, C., Vanhoucke, V., Ioffe, S., Shlens, J. & Wojna, Z. Rethinking the inception architecture for computer vision (2015). http://arxiv.org/abs/1512.00567

[41] Sokolova M, Lapalme G (2009). A systematic analysis of performance measures for classification tasks. Inf. Process. Manag..

